# The evolution of envelope function during coinfection with phylogenetically distinct human immunodeficiency virus

**DOI:** 10.1186/s12879-024-09805-z

**Published:** 2024-09-09

**Authors:** Shatha Omar, Zenda L. Woodman

**Affiliations:** 1 Department of Integrative Biomedical Sciences (IBMS), Division of Medical Biochemistry and Structural Biology, University of Cape Town, Cape Town, South Africa; 2 Present Address: Department of Biomedical Sciences, Division of Molecular Biology and Human Genetics, TB Genomics Group, Faculty of Medicine and Health Sciences, Stellenbosch University, Cape Town, South Africa

**Keywords:** HIV, Envelope, Coinfection; recombination, Viral fitness, Disease progression

## Abstract

**Background:**

Coinfection with two phylogenetically distinct Human Immunodeficiency Virus-1 (HIV-1) variants might provide an opportunity for rapid viral expansion and the emergence of fit variants that drive disease progression. However, autologous neutralising immune responses are known to drive Envelope (Env) diversity which can either enhance replicative capacity, have no effect, or reduce viral fitness. This study investigated whether in vivo outgrowth of coinfecting variants was linked to pseudovirus and infectious molecular clones’ infectivity to determine whether diversification resulted in more fit virus with the potential to increase disease progression.

**Results:**

For most participants, emergent recombinants displaced the co-transmitted variants and comprised the major population at 52 weeks postinfection with significantly higher entry efficiency than other co-circulating viruses. Our findings suggest that recombination within gp41 might have enhanced Env fusogenicity which contributed to the increase in pseudovirus entry efficiency. Finally, there was a significant correlation between pseudovirus entry efficiency and CD4 + T cell count, suggesting that the enhanced replicative capacity of recombinant variants could result in more virulent viruses.

**Conclusion:**

Coinfection provides variants with the opportunity to undergo rapid recombination that results in more infectious virus. This highlights the importance of monitoring the replicative fitness of emergent viruses.

**Supplementary Information:**

The online version contains supplementary material available at 10.1186/s12879-024-09805-z.

## Background

Human Immunodeficiency Virus-1 (HIV-1) transmission is usually due to a single variant followed by rapid diversification of *envelope* (*env*) through the selection of polymorphisms that enable evasion of autologous neutralising antibodies (nAb) which appear within weeks of infection [1, 2]. Therefore, escape from immune responses ensure that circulating variants continue to evolve, as illustrated by the emergence of a new HIV-1 subtype L [3]. This has direct consequences to vaccine and drug design and emphasises the need for continued epidemiological studies into the link between immune responses, *env* diversification and viral fitness.

Escape of variants from contemporaneous nAb over the course of the pandemic has resulted in the emergence of circulating variants with resistance to broadly neutralising antibodies (bnAb) [4]. Furthermore, natural selection has also selected for a more infectious virus with higher replicative capacity (RC) [5]. In general, escape mutations have been associated with a fitness cost [6, 7] but reversion and the introduction of compensatory mutations can restore the virus to its original fitness level and might even increase viral fitness [8, 9].

Recently, more transmissible viruses have been identified in north America which have been linked to increased viral load [10] and a highly virulent HIV strain was identified in Europe with increased viral fitness [11]. Furthermore, it has been shown that CRF19_cpx prevalent in Cuba is associated with rapid disease progression [12]. One mechanism could be that mutations in Env that confer resistance to bnAb allow for binding to alternative co-receptors, increasing the range of host cells susceptible to HIV infection [8].

Recently, a study on three individuals coinfected with two or more phylogenetically distinct HIV-1 variants detailed the longitudinal development of nAb specific for each variant [13]. They found that within the first year of infection, one variant was preferentially targeted after which neutralising immune responses shifted to another virus. In vitro neutralisation of Env pseudotyped virus (PSV) was associated with the decrease in in vivo frequency of the targeted variant but once responses waned, variant frequency seemed to be restored. In this study, we determined whether the change in in vivo variant frequency observed by Sheward *et al.* (2022) was associated with the entry efficiency (EE) of the Env clones, and whether the relationship was linked to disease progression [13].

## Materials and methods

### Ethics statement

Buffy coats were obtained from the Western Province blood service and as all donors were anonymous, there was no need to obtain informed consent. The protocol was approved by the University of Cape Town Ethics in Research Committee of the Faculty of Science, SFREC 003_2012.

### Study cohort

This study utilized stored PCR products of four participants: CAP37, CAP84, CAP137 and CAP267 that were generated as part of the CAPRISA 002 Acute Infection study, Durban, South Africa [14]. These participants were identified as coinfected as they were infected with two phylogenetically distinct variants prior to seroconversion as previously described [15]. CD4 + T cell counts were reported previously [16].

### Sequence analysis

A total of 295 single-genome amplification- (SGA-) derived *env* sequences (range = 6 to 30 sequences per time point) were generated as previously described [1] of four coinfected participants [15]. CAP84, CAP267 (typical progressors) and CAP137, CAP37 (rapid progressors), were analysed at enrolment [range: 1–6 weeks postinfection (wpi)] and approximately 12, 24 and 52 wpi to determine the frequency of variants over time [accession numbers: FJ443168- FJ443176, FJ443443- FJ443448, EF203963, EF203964, FJ443159- FJ443163, FJ443418- FJ443429 (www.ncbi.nlm.nih.gov)]. The disease progression status of CAP84, CAP37, CAP267 and CAP137 was assigned based on a decrease in CD4+ T cell levels to < 350 cells/µl within 2 years [16]. Master sequences used in Highlighter analysis (http://www.hiv.lanl.gov) represented the most phylogenetically distinct viruses circulating during early infection based on DNA distance (MEGA-5). The frequency of viral populations was determined relative to the total number of sequences at each time point (%). Recombination was determined by Recombination Identification Programme (RIP) analysis (http://www.hiv.lanl.gov).

### Envelope amplicons selection and cloning

SGA-derived *env* amplicons were chosen for cloning to represent co-transmitted and recombinant variants at each time point. The HIV-1 *env* gene was amplified using Phusion Hot Start DNA Polymerase (Thermo Scientific, USA) with primers EnvN (5’ CTG CCA ATC AGG GAA AGT AGC CTT GT 3’) (HXB2 K03455.1 numbering: 9145) and Env 1A-Rx (5’ CAC CGG CTT AGG CAT CTC CTA TAG CAG GAA GAA 3’) (HXB2 numbering: 5950) as described [1]. The resulting amplicons were cloned into the mammalian expression vectors pcDNA3.1D/V5-His-TOPO (Invitrogen) or pTarget (Promega, US) according to the manufacturer’s instructions. The sequences of the clones are available as supplementary data. Functionality of Env clones was tested by infecting TZM-bl cells with PSV and calculating the average relative light units (RLU) using a luciferase reporter assay as described in detail below.

### Entry efficiency

PSV were generated by co-transfecting HEK293T cells with *env* and pSG3∆Env HIV-1 backbone vectors (obtained through the NIH AIDS Reagent Program, Division of AIDS, NIAID, NIH from Drs. John C. Kappes and Xiaoyun Wu: HIV-1 SG3ΔEnv Non-infectious Molecular Clone) and viral titre was determined using p24 ELISA (Aalto Biosystems, Ireland) with the substrate CDP-Star/SapphireII (Applied Biosystems, USA). In a 96-well plate 10^4^ TZM-bl cells [NIH AIDS Reagent Program, Division of AIDS, NIAID, NIH: TZM-bl cells (Cat# 8129) from Dr. John C. Kappes, and Dr. Xiaoyun Wu] were infected with PSV normalized to 100 ng/ml of p24. RLU were measured using Bright-Glo Luciferase Assay System (Promega, USA) and a GloMax-Multi Microplate Multimode Reader (Promega, USA). EE of each clone was expressed relative to clone 1 (c1) of each participant.

### Generating infectious molecular clones

CAP137 and CAP267 infectious molecular clones (IMCs) were generated using a modified yeast gap-repair homologous recombination method as previously described [17, 18]. Briefly, full-length HIV-1 *env* was amplified from selected clones using the Phusion Hot Start Polymerase kit (Thermo Scientific, USA) with primers, Env IF (HXB2 numbering: 6202) (5’-AGA AAG AGC AGA AGA CAG TGG CAA TGA-3’) and Env IR (HXB2 numbering: 8791) (5’-TTT TGA CCA CTT GCC ACC CAT-3’). *Saccharomyces cerevisiae* (strain S288C) (Gift from Dr. Manish Sagar, Brigham and Women’s Hospital, Harvard Medical School, US) was made competent using 10% glycerol and transformed with a mixture of *env* PCR product (1 µg/µl) and linear pCMV-NL4-3-PBS/LTRΔGp160 (200 ng/µl). The shuttle vector, pCMV-NL4-3-PBS/LTRΔGp160 (3 µg), lacking *env* sequence between nucleotides 6346–8797 according to HXB2 numbering, was linearized with the FastDigest *Pac*I restriction enzyme (1U/ µl) (Thermo Scientific, USA). Transformed cells were grown and selected on 5-fluoro-1, 2, 3, 6-tetrahydro-2, 6-dioxo-4-pyrimidine carboxylic acid (5’FOA) and colonies were cultured before plasmid extraction (Zymo Research, USA). The recombinant plasmids were sequenced. Replication-competent viruses were generated by co-transfecting HEK293T cells with equivalent quantities of the recombinant plasmid and the helper plasmid (pCMV NL4-3 Gag4) [11]. Supernatants were collected 48 to 72 h after transfection, and tissue culture infectious dose (TCID50) was calculated based on Reed-Muench method [19].

### Replication assay in PBMCs

Peripheral blood monocytes (PBMCs) were isolated from HIV-negative donors using Ficoll-gradient centrifugation and activated with Interleukin-2 (IL-2) (200 U/ml) (Gentaur, Belgium) and phytohemagglutinin-P (0.5 µg/ml) (Remel, Thermo Scientific, USA) in RPMI 160 for 72 h at 37 °C and 5% CO_2_. Activated PBMCs (10^6^ cells/ml) were infected with 300 TCID50 of virus. Culture medium was collected on days 7, 10, and 14 postinfection, replaced with fresh medium and p24 concentration was determined using ELISA (Alto-Biosystems). IMC replication was compared between viruses by determining the slope of the graphs between days 0 and 7, 0 and 10, 0 and 14 [20]. The slope values of two independent measurements were averaged for each virus. NL4-3 HIV-1 [NIH AIDS Reagent Program, Division of AIDS, NIAID, and NIH: HIV-1 NL4-3 Infectious Molecular Clone (pNL4-3) from Dr. Malcolm Martin (Cat# 114)] provirus was used as a positive control and mock infection was used as a negative control.

### Env sensitivity to enfuvirtide

PSV was pre-incubated with five-fold serial dilution of the entry inhibitor drug Enfuvirtide (T20) (0.00032–5.0 µg/ml) [NIH AIDS Reagent Program (ARP), Division of AIDS, NIAID, NIH: T20] in DMEM for 1 h at 37 °C prior to infection of TZM-bl cells (10^4^ cells). Infections were incubated at 37 °C with 5% CO_2_ for 48 h and RLU determined as previously described [21]. The 50% inhibitory concentration (IC50) was determined using GraphPad Prism software 5.0 (CA, USA).

### Statistical analysis

GraphPad Prism 5.0 software (CA, USA) was used to perform all statistical analysis. One-way ANOVA with Bonferroni correction for multiple comparison was used to compare EE and replication capacity (RC) and Spearman correlation test was used to calculate the association between in vivo frequency and in vitro EE. Krustal-Wallis test with Dunn’s Multiple Comparison Test was used to compare the average pairwise DNA distance across all time points.

## Results

**Fig. 1:**
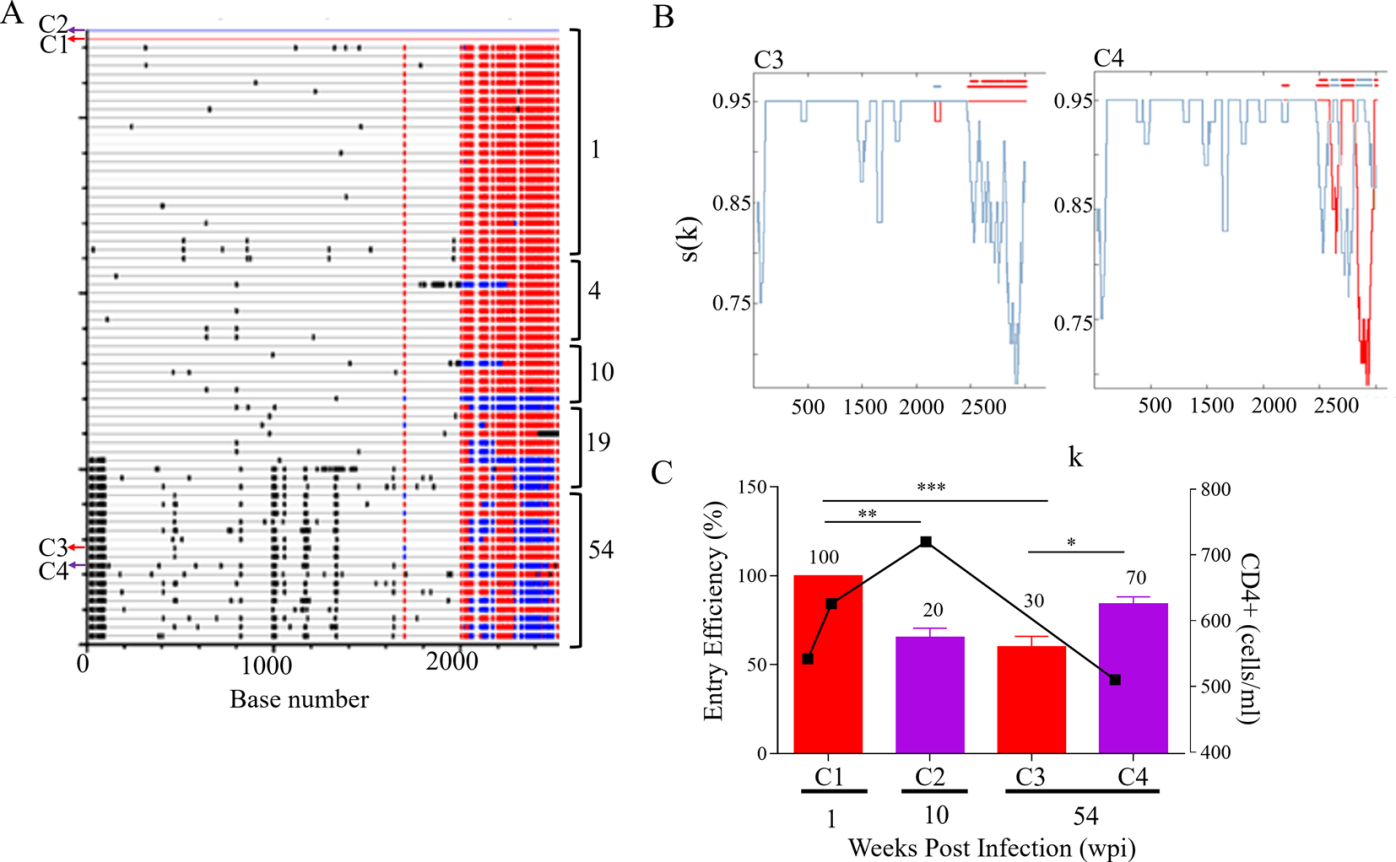
CAP84 *env* recombination and entry efficiency. (**A** ) The full-length *env* sequences of CAP84 were analysed using Highlighter (www.lanl.gov) with sequences from 1 weeks postinfection (wpi), 4, 10, 19 and 54 wpi compared to C1 (1 wpi) and C2 (4 wpi) master sequences. Sequences similar to C1 and C2 variants are shown in red and blue, respectively, while black lines indicate unique sequence not present in either master sequences. Sequences common to both master sequences are not coloured. The sequences cloned at 1, 10, and 54 wpi are indicated with arrows. (**B** ) RIP analysis of sequences representing recombinant Env at 54 wpi carried regions in gp41 that originated from C1 and C2 viruses. C1 is shown in red and C2 is indicated in blue and the top line is the query sequence. The x-axis (k) represents the query sequence position at the centre of the moving window of 400 bp. The y-axis, s(k), shows the similarity between the query sequence and C1 and C2. (**C** ) The entry efficiency (EE) of pseudovirus (PSV) representing C1 (red), and the recombinants (purple) were compared at 1, 10 and 54 wpi. PSV EE is shown relative to C1 (%) and represents the average of three independent biological repeats with error bars indicating standard deviation. The in vivo frequency (%) of each virus is indicated at the top of each bar. Decline in CD4 + T cell count is used as a marker of disease progression and shown by black squares on the right y-axis. CD4 + T cell counts were only included when data was available at the time points Env had been cloned or analysed. One-way ANOVA with Bonferroni correction for multiple comparisons was used for statistical analysis. (*p* ≤ 0.05: *, *p* ≤ 0.01: **, *p* ≤ 0.001: ***, and *p* ≤ 0.0001: ****)

**Fig. 2:**
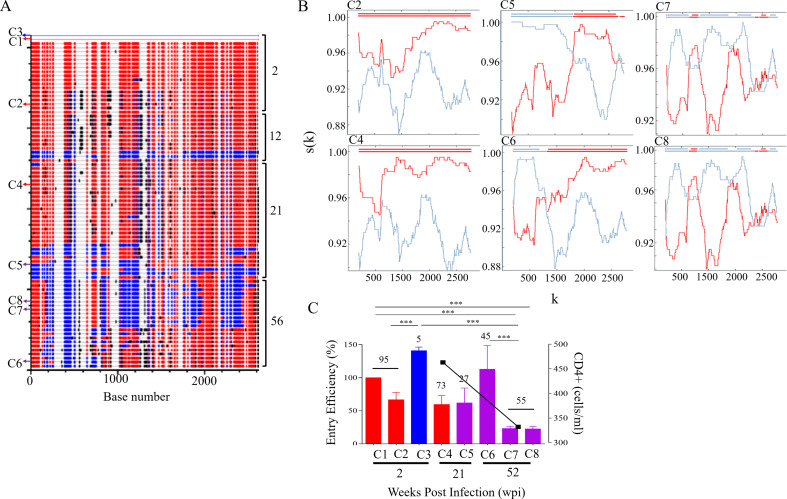
CAP37 Env recombination and entry efficiency. (**A** ) The full-length *env* sequence alignments of CAP37 were analysed using Highlighter (www.lanl.gov) with sequences from 2 week postinfection (wpi), 12, 21, and 56 wpi compared to C1 and C3 masters (both from 2 wpi). Sequences representing C1 and C3 viruses are shown in red and blue, respectively, while black lines indicate unique sequence not present in either master sequences. Sequences common to both master sequences are not coloured. The sequences cloned at 2, 21, and 56 wpi are indicated with arrows. (**B** ) RIP analysis of sequences representing recombinants at 21 and 56 wpi indicated two subpopulations: C5 and C6 were more similar to C1, whereas C7 and C8 had additional sequence from constant region 3 and gp41 from C3. C1 is shown in red and C3 is indicated in blue and the top line is the query sequence. The x-axis (k) represents the query sequence position at the centre of the moving window of 400 bp. The y-axis, s(k), shows the similarity between the query sequence and C1 and C3. (**C** ) The entry efficiency (EE) of pseudovirus (PSV) representing C1 (red), C3 (blue) and recombinants (purple) infecting CAP37 was compared at 2, 21 and 56 wpi. PSV EE is indicated relative to C1 and represents three independent biological repeats with error bars indicating standard deviation. The in vivo frequency (%) of each virus is indicated at the top of each bar. Decline in CD4 + T cell count is used as a marker of disease progression and shown by black squares on the right y-axis. CD4 + T cell counts were only included when data was available at the time points Env had been cloned or analysed. One-way ANOVA with Bonferroni correction for multiple comparisons was used for statistical analysis. (*p* ≤ 0.05: *, *p* ≤ 0.01: **, *p* ≤ 0.001: ***, and *p* ≤ 0.0001: ****)

**Fig. 3:**
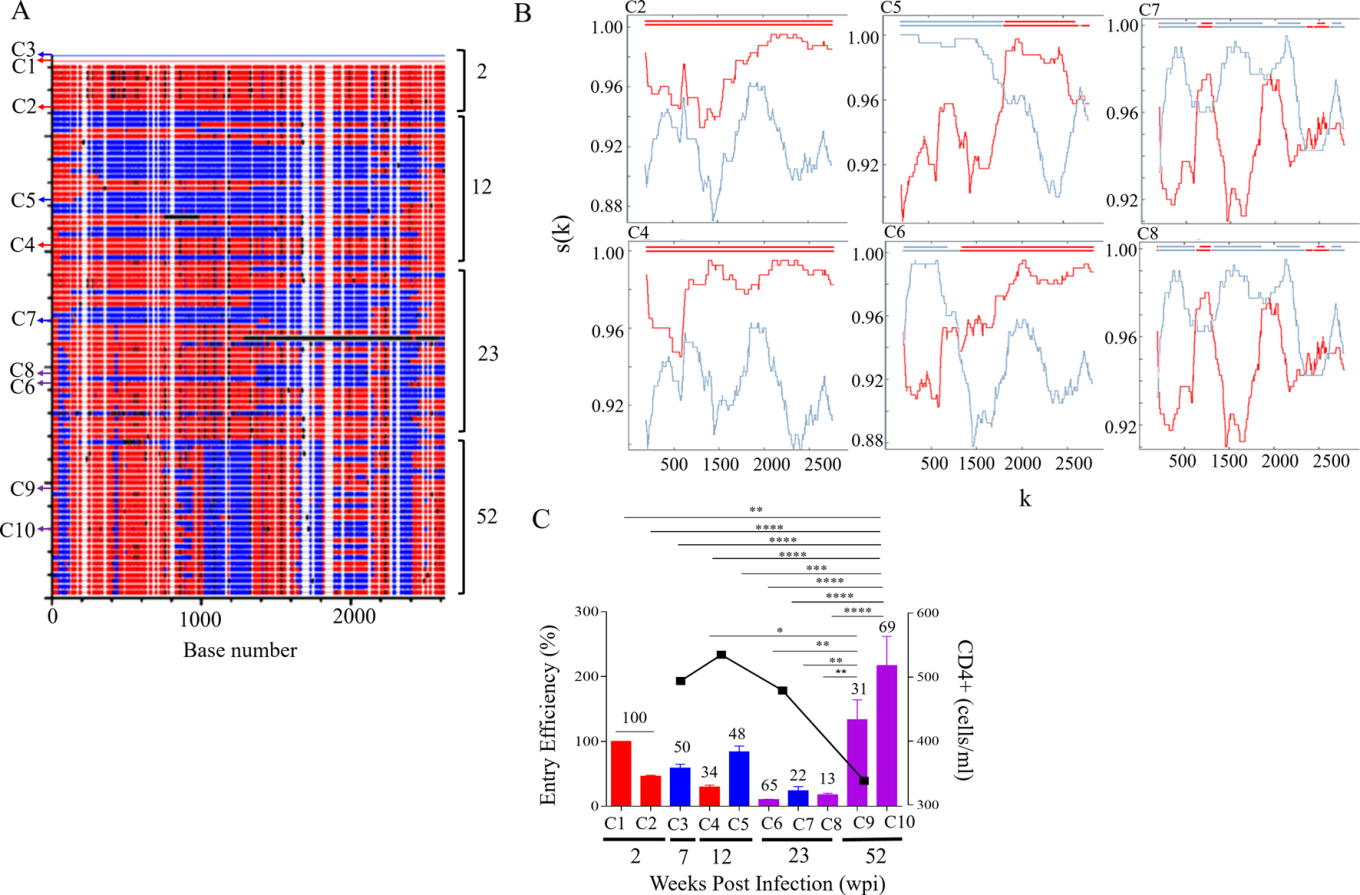
CAP137 *env* recombination and entry efficiency. (**A** ) The full-length *env* sequence alignments of CAP137 were analysed using Highlighter (www.lanl.gov) with sequences from 2 weeks postinfection (wpi), 7, 12, 23, and 52 wpi compared to C1 and C3 master sequences. Only C3 clone represents variants from 7 wpi. Sequences similar to C1 and C3 viruses are shown in red and blue, respectively, while black lines indicate unique sequence not present in either master sequences. Sequences common to both master sequences are not coloured. The sequences cloned at 2, 7, 12, 23, and 52 wpi are indicated with arrows. (**B** ) RIP analysis of sequences of CAP137 recombinant population at 52 wpi identified two sub-populations represented by C9 and C10. Both clones had common C3 sub-genomic regions but C10 carried additional sequence from C3 gp41. C1 is shown in red and C3 is indicated in blue and the top line is the query sequence. The x-axis (k) represents the query sequence position at the centre of the moving window of 400 bp. The y-axis, s(k), shows the similarity between the query sequence and C1 and C3. (**C** ) The entry efficiency (EE) of pseudovirus (PSV) representing C1 (red), C3 (blue) and recombinants (purple) infecting CAP137 was compared over time. PSV EE relative to C1 represents three independent biological repeats with error bars indicating standard deviation. The in vivo frequency (%) of each virus is indicated at the top of each bar. Decline in CD4 + T cell count is used as a marker of disease progression and shown by black squares on the right y-axis. CD4 + T cell counts were only included when data was available at the time points Env had been cloned or analysed. One-way ANOVA with Bonferroni correction for multiple comparisons was used for statistical analysis. (*p* ≤ 0.05: *, *p* ≤ 0.01: **, *p* ≤ 0.001: ***, and *p* ≤ 0.0001: ****)

**Fig. 4:**
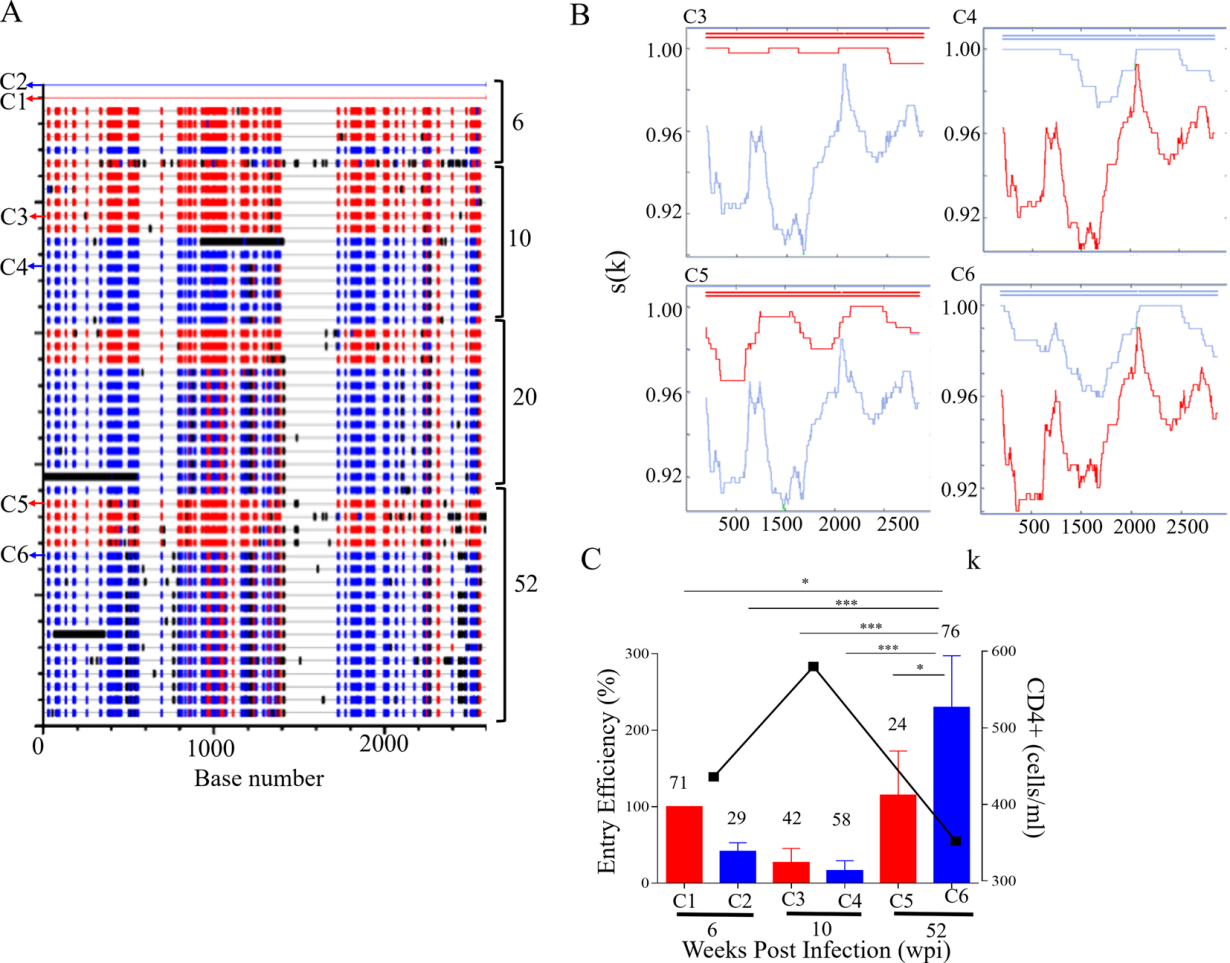
CAP267 *env* recombination and entry efficiency. (**A** ) The full-length *env* sequence alignments of CAP267 were analysed using Highlighter (www.lanl.gov) with sequences from 6 weeks postinfection (wpi), 10, 20, and 52 wpi compared to C1 and C2 master sequences. Sequences representing C1 and C2 viruses are shown in red and blue, respectively, while black lines indicate unique sequence not present in either master sequences. Sequences common to both master sequences are not coloured. The sequences cloned at different timepoints are indicated with arrows. (**B** ) RIP analysis of sequences at 10 and 52 wpi indicated that variants represented the cotransmitted viruses with no apparent recombination. C1 is shown in red and C2 is indicated in blue and the top line is the query sequence. The x-axis (k) represents the query sequence position at the centre of the moving window of 400 bp. The y-axis, s(k), shows the similarity between the query sequence and C1 and C2. (**C** ) The entry efficiency (EE) of pseudovirus (PSV) representing virus C1 (red), and C2 (blue) infecting CAP267 was compared at 6, 10 and 52 wpi. PSV EE relative to C1 represents three independent biological repeats with error bars indicating standard deviation. The in vivo frequency (%) of each virus is indicated at the top of each bar. Decline in CD4 + T cell count is used as a marker of disease progression and shown by black squares on the right y-axis. CD4 + T cell counts were only included when data was available at the time points Env had been cloned or analysed. One-way ANOVA with Bonferroni correction for multiple comparisons was used for statistical analysis. (*p* ≤ 0.05: *, *p* ≤ 0.01: **, *p* ≤ 0.001: ***, and *p* ≤ 0.0001: ****)

As previously indicated CAP37, CAP137 and CAP267 were infected with highly diverse variants [13] whereas variants infecting CAP84 had a maximum DNA distance of only 3% within Env. Recombination is a common feature of coinfections [22] and we found that recombination occurred in CAP37, CAP84 and CAP137 mainly within the constant 3 (C3) region and gp41 although there was evidence that recombination also occurred within other regions such as the signal peptide (Figs. 1, 2, 3 and 4). To determine whether emergent recombinants had increased Env EE, we compared PSV EE of 8, 4, 10 and 6 functional Env clones representing transmitted and recombinant variants infecting CAP37, CAP84, CAP37 and CAP267, respectively, across the first year of infection (Table 1).

**Table 1:** Envelope clones representing viral populations over time in coinfected individuals

Participant ID	Time postinfection (weeks)	^a^Clone ID
CAP37	2	C1; C2; C3
	21	C4; C5
	56	C6; C7; C8
CAP84	1	C1
	10	C2
	54	C3; C4
CAP137	2	C1; C2; C3
	12	C4; C5
	23	C6; C7; C8
	52	C9; C10
CAP267	6	C1; C2
	10	C3; C4
	52	C5; C6

^a^ In text, clone ID can be written as: Participant ID_Clone ID

### Coinfection with one detectable variant: CAP84

Initially, CAP84 was identified as coinfected with two phylogenetically distinct strains at 1 wpi using heteroduplex analysis of the constant 2–3 (C2-C3) region [15] but according to full-length *env* analysis CAP84 was infected with a homogenous viral population. This suggests that the second transmitted variant was at a frequency too low to detect by single genome amplification and sequencing. Variants with sequence changes in gp41 were detected at 4 wpi and by 54 wpi distinct gp41 recombinants had emerged that dominated the viral population (Fig. 1A and B). Four *env* clones were generated that represented viruses at 1, 10 and 54 wpi. There was a decrease in frequency and EE of the transmitted variant with concomitant outgrowth of recombinants with higher EE (Fig. 1C). At 54 wpi the outgrowth of variants recombined within gp41 coincided with low CD4 + T cell count.

An early study showed that autologous nAb to the major co-transmitted variant emerged at 23 wpi [23] which coincided with the outgrowth of the recombinant variants (Fig. 1A). Although EE was restored at 54 wpi, recombination did not increase EE relative to the transmitted variant. Interestingly, the similar EE at 1 and 54 wpi coincided with similar CD4 + T cell counts suggesting an association between Env function and virulence.

### Coinfection with highly diverse variants: CAP37 and CAP137

CAP37 was infected with highly diverse co-transmitted variants at 2 wpi. The major population at this timepoint was replaced by recombinants after 21 wpi. The recombined variants continued to evolve until two major sub-populations emerged with similar frequency (Fig. 2A and B). There was no apparent relationship between frequency and Env EE with the two recombinant subgroups occurring at similar frequencies despite one group having 4-fold significantly higher EE than the other (Fig. 2C). CAP37 Env variants shared sub-genomic regions which lead to cross-neutralisation of co-transmitted and recombinant variants [13], suggesting that all variants were under similar immune pressure. However, by 52 wpi, one recombinant population, represented by 137C6 (Table 1) had evolved to higher EE than the other which coincided with a drop in CD4 + T cells.

Within 7 weeks of infection, CAP137 was infected with two phylogenetically distinct viruses but by 23 wpi, the dominant co-transmitted strain was replaced by recombinant variants (Fig. 3A and B). Sheward *et al.* (2022) found that nAb targeted distinct epitopes within the parent sequences and immune responses to 137C2 were delayed till 19 wpi [13]. By 52 wpi CAP137 was infected with two sub-populations representing recombinants that differed in gp41 but carried similar C3 and variable 4 (V4) regions from 137C2 gp120 (Fig. 3B). The recombinant carrying 137C2 gp41 was the dominant variant at 52 wpi with the highest EE (Fig. 3C). The sequential targeting of the co-transmitted variants enabled rapid recombination that facilitated immune escape and reduced EE. However, further selection resulted in the outgrowth of recombinants with high EE.

### Coinfection with recombinant variants: CAP267

CAP267 was initially infected with two populations that shared sub-genomic regions likely due to recombination (Fig. 4A). The frequency of the major population at 6 wpi declined over time, with the concomitant rise in the second viral population, becoming the dominant population at 52 wpi (Fig. 4A and C). There was no apparent recombination between the parent viruses over 12 months of infection (Fig. 4B). When the EE of six Env clones were compared, the dominant variant at 6 wpi had the highest EE which declined over the course of infection. The second variant, on the other hand, not only increased in frequency but also had enhanced EE (Fig. 4C). Similar to CAP137, nAb responses to one variant occurred weeks after the other which suggested immune interference. Preferential targeting by autologous nAb switched from one variant to another which coincided with decreased frequency. Notably, the dominant variant at 52 wpi was not targeted [13] which corresponded to increased EE. On average, Env EE tracked changes in frequency for CAP267.

### Envelope contributes to viral replicative fitness

For CAP84, CAP137 and CAP267, the viral population with the highest frequency at 1 year postinfection also had significantly higher EE than the less dominant variant at the same time point. Therefore, overall outgrowth of variants, either over 12 months of infection or within the same time point could be due to enhanced Env EE. However, there was no significant association between in vivo variant frequency and in vitro PSV EE (*p* = 0.37, *r* = 0.19).

**Fig. 5:**
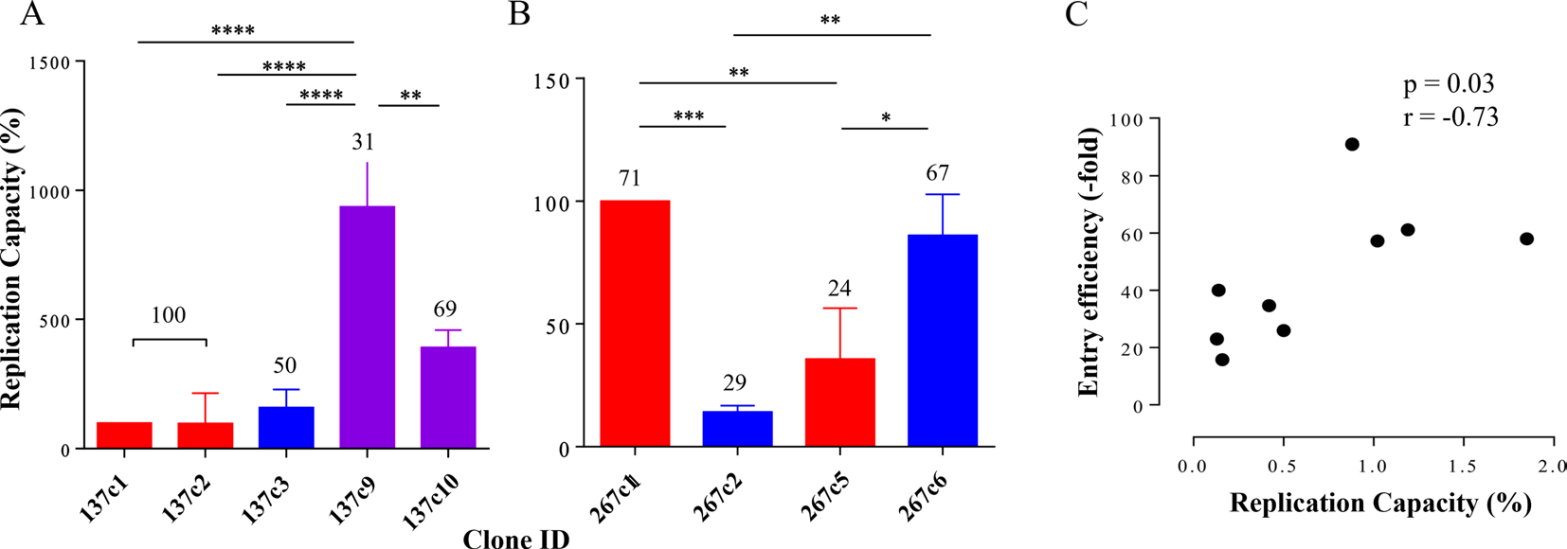
Relationship between Env function and variant frequency. The replication capacity (RC) of (**A** ) CAP137 and (**B** ) CAP267 chimeric infectious molecular clones (IMC) was compared at the first timepoint and 52 wpi. Slope values were used to calculate the mean and standard deviation of two PBMC donors. Entry efficiency (EE) and RC are indicated relative to C1, the major transmitted variant. The in vivo frequency (%) of each virus is indicated at the top of each bar. One-way ANOVA with Bonferroni correction for multiple comparisons was used for statistical analysis. (*p* ≤ 0.05: *, *p* ≤ 0.01: **, *p* ≤ 0.001: ***, and *p* ≤ 0.0001: ****). (**C** ) Correlation analysis was carried out between PSV EE and RC. PSV EE was normalised to the cell only control and RC was expressed as the mean of the slope of two independent experiments relative to C1. Spearman r test was used to indicate a negligible, weak, moderate, high and very high relationship based on previous reports [39], p value of < 0.05 was considered significant

PSV EE is limited to a single round of infection and might not be representative of in vivo virus propagation. Provine *et al.* (2009) reported that IMC phenotype was not always similar to that of corresponding PSV, [24]potentially due to differences between producing cells: HEK293T cells and PBMCs [24–27]. We therefore constructed pNL4.3 IMCs carrying the Env clones to confirm that our PSV EE data represented viral replication. We determined the in vitro RC of chimeric IMCs for CAP137 and CAP267. For both participants, IMCs representing the dominant viral population at 52 wpi had higher RC than the major transmitted variant, indicating that viruses better able to replicate emerged over time (Fig. 5A and B). Although, the RC of IMCs did not mimic the EE of all clones there was a significant correlation (*p* = 0.03, *r* = 0.7) between the RC of chimeric IMCs in PBMCs and their corresponding PSV EE in TZM-bl cells (Fig. 5C).

### Fusogenicity might drive changes in Env-driven entry efficiency and replication

**Fig. 6:**
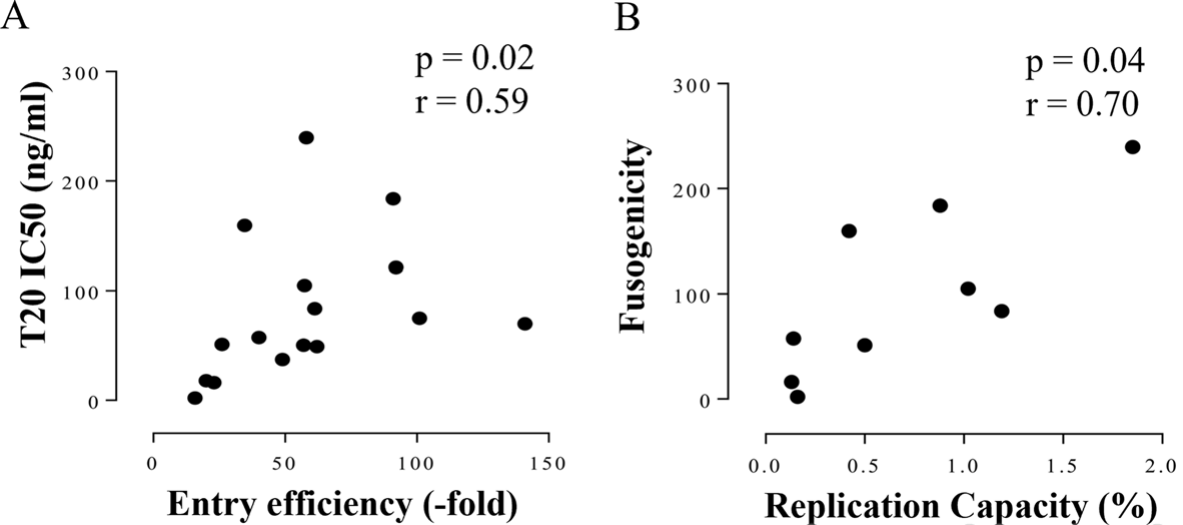
Correlation between Env function and Fusogenicity. The association between fusogenicity as measured by T20 sensitivity using TZM-bl cells and corresponding (**A** ) Env entry efficiency (EE) of pseudovirus (PSV) and (**B** ) replication capacity (RC) of infectious molecular clones were analysed using Spearman r correlation test. T20 IC50 (ng/ml) was used as an indicator of Env fusogenicity. PSV EE was normalised to the cell only control and RC was expressed as the mean of the slope derived from replication kinetics of two independent experiments relative to C1. Correlation co-efficient (r) was indicative of a negligible, weak, moderate, high and very high relationship based on previous reports [39], p value of < 0.05 was considered significant

Virus entry can be blocked by T20 inhibition of gp41, the subunit responsible for virus-host membrane fusion. Therefore, T20 IC50 has been used as a surrogate marker for PSV fusogenicity [21, 28]. PSV will become less sensitive to T20 inhibition as the fusogenicity of Env increases [28–31]. As three participants were infected with variants with recombination in gp41, we determined whether increased PSV EE and IMC RC were associated with changes in T20 IC50. Correlation analysis showed that variants with higher EE and RC, also had higher fusogenicity (Fig. 6). This suggested that changes in PSV EE could be due to changes in gp41 fusogenicity which could then impact variant frequency.

### Envelope fitness and disease progression

**Fig. 7:**
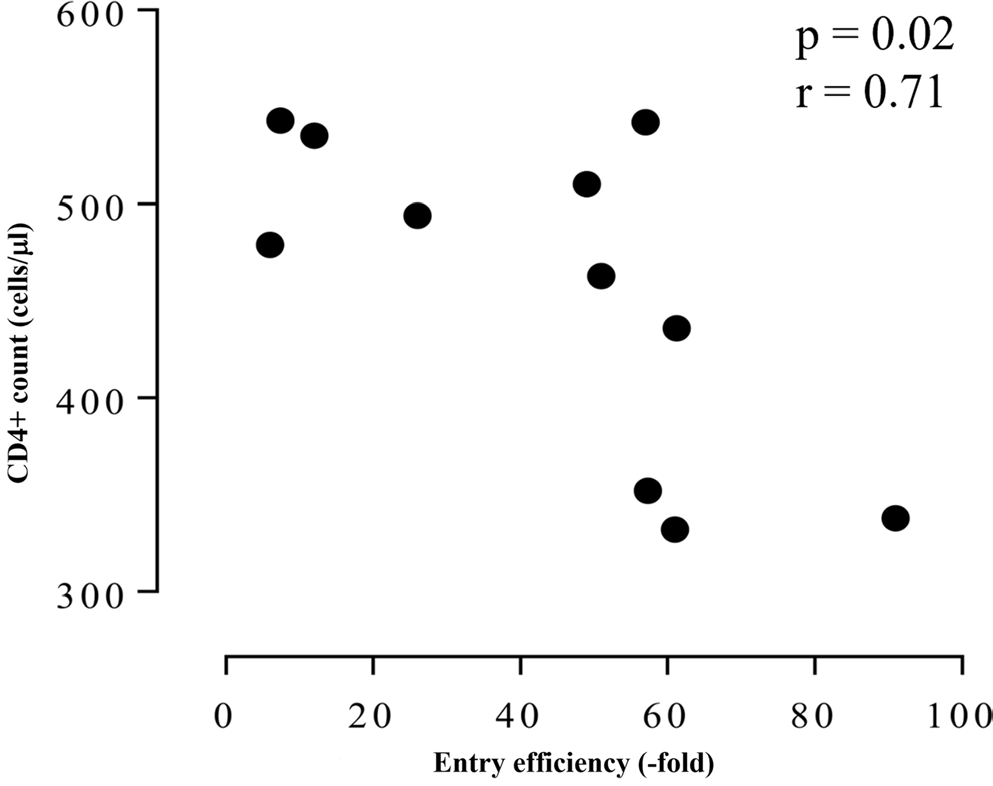
Correlation between Env function and disease progression. Correlation analysis was carried out between pseudovirus (PSV) entry efficiency (EE) and CD4 + T cell count. PSV EE was normalised to the cell only control and indicated as fold change. Only the EE of the virus with the highest frequency was compared to CD4 + T cell counts for all participants. Spearman r test was used to indicate a negligible, weak, moderate, high and very high relationship based on previous reports [39], p value of < 0.05 was considered significant

Env EE was reported to be associated with disease progression indicators: viral load (VL) and CD4 + T cell count [32–35]. For all participants, there was a decline in CD4 + T cell count over the first year of infection (Figs. 1, 2, 3 and 4) and there was a significant correlation (*p* = 0.046, *r* = -0.59) between CD4 + T cell loss and increased PSV EE. The association became more pronounced when CD4 + T cell count was compared to the EE of only the dominant variant (*p* = 0.02, *r* = -0.71) (Fig. 7), suggesting that viruses had become more virulent over time.

## Discussion

Phylogenetic analysis of the highly variable C2-C3 Env region identified nineteen coinfected individuals [15] and this study focussed on four study participants: CAP37, CAP137, CAP267 and CAP84. Sheward *et al.* (2022) showed that the frequency of variants infecting CAP37, CAP137 and CAP267 varied according to the specificity of autologous nAb responses [13]. We aimed to investigate whether changes in Env function corresponded to in vivo outgrowth of variants and whether variation in EE was associated with fluctuations in CD4 + T cell count as a proxy for virulence.

HIV escape from immune responses is associated with decreased RC [6, 7] although the introduction of compensatory mutations not only restores viral fitness, it can also lead to increased replication [8, 9]. A number of studies have shown that natural selection has enriched for circulating variants resistant to neutralisation with increased transmissibility and virulence [4, 5, 10–12]. When neutralisation of two or more phylogenetically distinct HIV-1 variants, isolated from coinfected individuals, were compared [13], nAb preferentially targeted one variant over another. The frequency of the targeted variant decreased until escape from neutralisation alleviated immune pressure and allowed for continued viral replication. We hypothesised that escape from Env-specific nAb did not only restore variant frequency but selected for fitter variants.

CAP267 and CAP84 were coinfected at 1 wpi with variants that shared sub-genomic regions suggesting the co-transmission of recombinants. CAP84 gp41 recombinants continued to emerge over the course of infection whereas co-transmitted CAP267 variants remained mostly unchanged. On the other hand, parent viruses were detected at 2 wpi for CAP37 and CAP137 and recombinants were only detected at 12 wpi. Recombinant viruses, whether transmitted or emergent, were dominant at 52 wpi for all participants. Recombination contributed to the high sequence diversity and most likely, subsequent enhanced viral fitness [32, 34, 36, 37]. Point mutations play a significant role in viral fitness, and subsequent recombination would ensure the rapid establishment and fixation of advantageous polymorphisms, aiding rapid immune escape and improved RC [38]. CAP267 was infected with recombinant variants that did not undergo further recombination, suggesting advantageous point mutations were either fixed prior to co-transmission or that the accumulation of new polymorphisms during infection were sufficient to enhance EE and RC.

Compared to the co-transmitted founders, there was a decrease in EE for all viruses over the first few months, after which, EE was restored. This supports the notion that immune escape decreases viral fitness but reversion mutations compensates for this loss [9]. The dominant variants infecting CAP137 and CAP267 at 52 wpi had significantly higher PSV EE compared to the co-transmitted variants and the replication capacity of the corresponding IMCs showed a similar trend, suggesting that RC played an important role in the competitive ability of variants at 12 mpi for these two participants.

Sheward *et al.* (2022) suggested alternative types of neutralisation: CAP37, cross-neutralisation; CAP137, interference/additive responses and CAP267, interference, rationalising that the extent of diversity and distribution of similar sub-genomic regions between phylogenetically distinct variants elicited varying immune responses [13]. It is possible that the type of autologous nAb response might play a role in the emergence of fitter viruses over the first year of infection.

Except for CAP37, the dominant variant at 52 wpi either had higher EE than the co-transmitted founders or co-circulating viruses. For CAP37, there was no clear dominant population at 52 wpi and both sub-populations had either the same or poorer EE than the initial virus at 2 wpi. Co-transmitted and recombinant variants infecting CAP37 were potently neutralised by antibodies to shared regions. It is possible that immune pressure did not allow for the emergence of recombinants with higher entry efficiency. On the contrary, for CAP267 and CAP137, neutralisation of one variant seemed to interfere with the immune response to the other. The delay in immune response to one co-transmitted variant might have enabled outgrowth of the other population, allowing for rapid sampling of sequences and the selection of the most beneficial combination of polymorphisms or sub-genomic regions. However, without analysing the immune response to variants from later samples and confirming whether sequence changes contributed to both immune escape and increased EE, no conclusions can be made. Another limitation of the study is that the selected clones might not represent the fitness of the circulating population at each time point as small shifts in recombination breakpoints and point mutations between strains could impact Env function. However, as the sequence of each clone was similar to consensus at each timepoint, it is likely that recombination selected for variants with enhanced EE and RC. The apparent rapid recombination within gp41 of CAP37, CAP84 and CAP137 could suggest that fusogenicity might be an important mechanism for enhancing virus infectivity [32, 39–42]. To investigate the relationship between Env fusogenicity and virus infectivity, we determined the sensitivity of some clones to T20 and found that there was a significant correlation between IC50 values and PSV EE and IMC RC. Rapid recombination could promote viral replication and outgrowth by selecting for variants with high fusogenicity.

Of the four participants, two were reported to be rapid progressors (CAP37 and CAP137) and although CAP267 was classified as a typical progressor, her CD4 + T cell count dropped to below 350 cells/µl within two years. Furthermore, there was a significant association between PSV EE and CD4 + T cell levels, suggesting that those participants infected with variants with high Env-driven RC might have enhanced disease progression.

This study showed that Env clones evolve to higher fitness in coinfected individuals which, in general, seems to be associated with increased frequency, EE, fusogenicity and RC of variants at 12 mpi. There did not seem to be a consistent link to diversity at transmission most likely due to the impact of immune responses on viral titres. The association between Env fitness and CD4 + T cell count suggests that the interplay between variant RC and immune responses might select for more virulent strains. This emphasises the need to continue monitoring the virulence of circulating HIV 1 variants to prevent the spread of emergent, more pathogenic strains. Furthermore, it is possible that vaccines able to neutralise circulating variants might drive the evolution of escape variants to higher fitness levels, suggesting that epitope selection and immunogen design could have detrimental consequences.

## Electronic supplementary material

Below is the link to the electronic supplementary material.

Supplementary Material 1

## Data Availability

The datasets generated and/or analyzed during the current study are available in the FigShare repository: https://doi.org/10.25375/uct.26539267.v1.

## Abbreviations

ARP: AIDS Reagent Program
bnAb: Broadly neutralising antibodies
T20: Enfuvirtide
EE: Entry efficiency
*env*: Envelope gene
Env: Envelope protein
5’FOA: on 5-fluoro-1, 2, 3, 6-tetrahydro-2, 6-dioxo-4-pyrimidine carboxylic acid
HIV-1: Human Immunodeficiency Virus-1
IMC: Infectious molecular clones
IL-2: Interleukine-2
PBMCs: Peripheral blood monocytes
nAb: Neutralising antibodies
PSV: Pseudovirus
RLU: Relative light units
RC: Replication capacity
SGA: Single genome amplification
TCID50: Tissue culture infectious dose
IC50: The 50 % inhibitory concentration
Wpi: Weeks postinfection
VL: Viral load

## References

[1] Abrahams M-R, Anderson JA, Giorgi EE, Seoighe C, Mlisana K, Ping L-H, et al. Quantitating the multiplicity of infection with human immunodeficiency virus type 1 subtype C reveals a non-poisson distribution of transmitted variants. J Virol. 2009;83(8):3556–67. 19193811 10.1128/JVI.02132-08 PMC2663249

[2] Frost SD, Wrin T, Smith DM, Pond SLK, Liu Y, Paxinos E, et al. Neutralizing antibody responses drive the evolution of human immunodeficiency virus type 1 envelope during recent HIV infection. Proc Natl Acad Sci. 2005;102(51):18514–9. 10.1073/pnas.0504658102 PMC1310509 16339909

[3] Yamaguchi J, Vallari A, McArthur C, Sthreshley L, Cloherty GA, Berg MG, et al. Brief report: complete genome sequence of CG-0018a-01 establishes HIV-1 subtype L. J Acquir Immune Defic Syndr. 2020;83(3):319–22. 31693506 10.1097/QAI.0000000000002246 PMC7012332

[4] Bouvin-Pley M, Morgand M, Moreau A, Jestin P, Simonnet C, Tran L, et al. Evidence for a continuous drift of the HIV-1 species towards higher resistance to neutralizing antibodies over the course of the epidemic. PLoS Pathog. 2013;9(7):e1003477. 23853594 10.1371/journal.ppat.1003477 PMC3701719

[5] Bouvin-Pley M, Beretta M, Moreau A, Roch E, Essat A, Goujard C, et al. Evolution of the envelope glycoprotein of HIV-1 clade B toward higher infectious properties over the course of the epidemic. J Virol. 2019;93(6):01171–18. 10.1128/jvi. 10.1128/jvi PMC6401426 30567994

[6] Lynch RM, Wong P, Tran L, O’Dell S, Nason MC, Li Y, et al. HIV-1 fitness cost associated with escape from the VRC01 class of CD4 binding site neutralizing antibodies. J Virol. 2015;89(8):4201–13. 25631091 10.1128/JVI.03608-14 PMC4442379

[7] Sather DN, Carbonetti S, Kehayia J, Kraft Z, Mikell I, Scheid JF, et al. Broadly neutralizing antibodies developed by an HIV-positive elite neutralizer exact a replication fitness cost on the contemporaneous virus. J Virol. 2012;86(23):12676–85. 22973035 10.1128/JVI.01893-12 PMC3497623

[8] Marichannegowda MH, Song H. Immune escape mutations selected by neutralizing antibodies in natural HIV-1 infection can alter coreceptor usage repertoire of the transmitted/founder virus. Virology. 2022;568:72–6. 35144109 10.1016/j.virol.2022.01.010 PMC9305671

[9] Nagaraja P, Alexander HK, Bonhoeffer S, Dixit NM. Influence of recombination on acquisition and reversion of immune escape and compensatory mutations in HIV-1. Epidemics. 2016;14:11–25. 26972510 10.1016/j.epidem.2015.09.001

[10] Wertheim JO, Oster AM, Switzer WM, Zhang C, Panneer N, Campbell E, et al. Natural selection favoring more transmissible HIV detected in United States molecular transmission network. Nat Commun. 2019;10(1):5788. 31857582 10.1038/s41467-019-13723-z PMC6923435

[11] Wymant C, Bezemer D, Blanquart F, Ferretti L, Gall A, Hall M, et al. A highly virulent variant of HIV-1 circulating in the Netherlands. Science. 2022;375(6580):540–5. 35113714 10.1126/science.abk1688

[12] Kouri V, Khouri R, Alemán Y, Abrahantes Y, Vercauteren J, Pineda-Peña A-C, et al. CRF19_cpx is an evolutionary fit HIV-1 variant strongly associated with rapid progression to AIDS in Cuba. EBioMedicine. 2015;2(3):244–54. 26137563 10.1016/j.ebiom.2015.01.015 PMC4484819

[13] Sheward DJ, Hermanus T, Murrell B, Garrett N, Abdool Karim SS, Morris L, et al. HIV Coinfection provides insights for the design of Vaccine cocktails to elicit broadly neutralizing antibodies. J Virol. 2022;96(14):e00324–22. 35758668 10.1128/jvi.00324-22 PMC9327685

[14] van Loggerenberg F, Mlisana K, Williamson C, Auld SC, Morris L, Gray CM, et al. Establishing a cohort at high risk of HIV infection in South Africa: challenges and experiences of the CAPRISA 002 acute infection study. PLoS ONE. 2008;3(4):e1954. 18414658 10.1371/journal.pone.0001954 PMC2278382

[15] Woodman Z, Mlisana K, Treurnicht F, Abrahams M-R, Thebus R, Karim SA, et al. Short communication decreased incidence of dual infections in South African subtype C-infected women compared to a cohort ten years earlier. AIDS Res Hum Retroviruses. 2011;27(11):1167–72. 21198409 10.1089/aid.2010.0162 PMC3206740

[16] Mlisana K, Werner L, Garrett NJ, McKinnon LR, Van Loggerenberg F, Passmore J-AS, et al. Rapid disease progression in HIV-1 subtype C–infected South African women. Clin Infect Dis. 2014;59(9):1322–31. 25038116 10.1093/cid/ciu573 PMC4271037

[17] Chatziandreou N, Arauz AB, Freitas I, Nyein PH, Fenton G, Mehta SH, et al. Sensitivity changes over the course of infection increases the likelihood of resistance against fusion but not CCR5 receptor blockers. AIDS Res Hum Retroviruses. 2012;28(12):1584–93. 22650962 10.1089/aid.2011.0319 PMC3505054

[18] Dudley DM, Gao Y, Nelson KN, Henry KR, Nankya I, Gibson RM, et al. A novel yeast-based recombination method to clone and propagate diverse HIV-1 isolates. Biotechniques. 2009;46(6):458–67. 19480640 10.2144/000113119

[19] Reed L, Muench H. A simple method of estimating fifty per cent endpoints. Am J Hygiene. 1938;27:493–7.

[20] Weber J, Vazquez AC, Winner D, Rose JD, Wylie D, Rhea AM et al. Novel method for simultaneous quantification of phenotypic resistance to maturation, protease, reverse transcriptase, and integrase HIV inhibitors based on 3′ Gag (http://p2/p7/p1/p6)/PR/RT/INT-recombinant) viruses: a useful tool in the multitarget era of antiretroviral therapy. Antimicrobial agents and chemotherapy. 2011;55(8):3729-42. 10.1128/AAC.00396-11 PMC3147656 21628544

[21] Reeves JD, Lee F-H, Miamidian JL, Jabara CB, Juntilla MM, Doms RW. Enfuvirtide resistance mutations: impact on human immunodeficiency virus envelope function, entry inhibitor sensitivity, and virus neutralization. J Virol. 2005;79(8):4991–9. 15795284 10.1128/JVI.79.8.4991-4999.2005 PMC1069568

[22] Charpentier C, Nora T, Tenaillon O, Clavel F, Hance AJ. Extensive recombination among human immunodeficiency virus type 1 quasispecies makes an important contribution to viral diversity in individual patients. J Virol. 2006;80(5):2472–82. 16474154 10.1128/JVI.80.5.2472-2482.2006 PMC1395372

[23] Gray E, Moore P, Choge I, Decker J, Bibollet-Ruche F, Li H, et al. Neutralizing antibody responses in acute human immunodeficiency virus type 1 subtype C infection. J Virol. 2007;81(12):6187–96. 17409164 10.1128/JVI.00239-07 PMC1900112

[24] Provine NM, Puryear WB, Wu X, Overbaugh J, Haigwood NL. The infectious molecular clone and pseudotyped virus models of human immunodeficiency virus type 1 exhibit significant differences in virion composition with only moderate differences in infectivity and inhibition sensitivity. J Virol. 2009;83(17):9002–7. 19535443 10.1128/JVI.00423-09 PMC2738156

[25] Herrera C, Klasse PJ, Michael E, Kake S, Barnes K, Kibler CW, et al. The impact of envelope glycoprotein cleavage on the antigenicity, infectivity, and neutralization sensitivity of Env-pseudotyped human immunodeficiency virus type 1 particles. Virology. 2005;338(1):154–72. 15932765 10.1016/j.virol.2005.05.002

[26] Murakami T, Freed EO. The long cytoplasmic tail of gp41 is required in a cell type-dependent manner for HIV-1 envelope glycoprotein incorporation into virions. Proc Natl Acad Sci. 2000;97(1):343–8. 10618420 10.1073/pnas.97.1.343 PMC26665

[27] Pugach P, Marozsan AJ, Ketas TJ, Landes EL, Moore JP, Kuhmann SE. HIV-1 clones resistant to a small molecule CCR5 inhibitor use the inhibitor-bound form of CCR5 for entry. Virology. 2007;361(1):212–28. 17166540 10.1016/j.virol.2006.11.004 PMC1892195

[28] Cavrois M, Neidleman J, Santiago ML, Derdeyn CA, Hunter E, Greene WC. Enhanced fusion and virion incorporation for HIV-1 subtype C envelope glycoproteins with compact V1/V2 domains. J Virol. 2014;88(4):2083–94. 24335304 10.1128/JVI.02308-13 PMC3911571

[29] Etemad B, Fellows A, Kwambana B, Kamat A, Feng Y, Lee S, et al. Human immunodeficiency virus type 1 V1-to-V5 envelope variants from the chronic phase of infection use CCR5 and fuse more efficiently than those from early after infection. J Virol. 2009;83(19):9694–708. 19625411 10.1128/JVI.00925-09 PMC2748008

[30] Lobritz MA, Marozsan AJ, Troyer RM, Arts EJ. Natural variation in the V3 crown of human immunodeficiency virus type 1 affects replicative fitness and entry inhibitor sensitivity. J Virol. 2007;81(15):8258–69. 17522224 10.1128/JVI.02739-06 PMC1951322

[31] Reeves JD, Gallo SA, Ahmad N, Miamidian JL, Harvey PE, Sharron M et al. Sensitivity of HIV-1 to entry inhibitors correlates with envelope/coreceptor affinity, receptor density, and fusion kinetics. Proceedings of the National Academy of Sciences. 2002;99(25):16249-54. 10.1073/pnas.252469399 PMC138597 12444251

[32] Gordon K, Omar S, Nofemela A, Bandawe G, Williamson C, Woodman Z. A recombinant variant with increased envelope Entry Efficiency emerged during early infection of an HIV-1 Subtype C Dual Infected Rapid Progressor. AIDS Res Hum Retroviruses. 2016;32(3):303–10. 25905681 10.1089/aid.2014.0100 PMC4779978

[33] Lassen KG, Lobritz MA, Bailey JR, Johnston S, Nguyen S, Lee B, et al. Elite suppressor–derived HIV-1 envelope glycoproteins exhibit reduced entry efficiency and kinetics. PLoS Pathog. 2009;5(4):e1000377. 19360131 10.1371/journal.ppat.1000377 PMC2661022

[34] Sagar M, Lavreys L, Baeten JM, Richardson BA, Mandaliya K, Chohan BH, et al. Infection with multiple human immunodeficiency virus type 1 variants is associated with faster disease progression. J Virol. 2003;77(23):12921–6. 14610215 10.1128/JVI.77.23.12921-12926.2003 PMC262567

[35] Troyer RM, Collins KR, Abraha A, Fraundorf E, Moore DM, Krizan RW, et al. Changes in human immunodeficiency virus type 1 fitness and genetic diversity during disease progression. J Virol. 2005;79(14):9006–18. 15994794 10.1128/JVI.79.14.9006-9018.2005 PMC1168764

[36] Cornelissen M, Pasternak AO, Grijsen ML, Zorgdrager F, Bakker M, Blom P, et al. HIV-1 dual infection is associated with faster CD4 + T-cell decline in a cohort of men with primary HIV infection. Clin Infect Dis. 2012;54(4):539–47. 22157174 10.1093/cid/cir849

[37] Templeton AR, Kramer MG, Jarvis J, Kowalski J, Gange S, Schneider MF, et al. Multiple-infection and recombination in HIV-1 within a longitudinal cohort of women. Retrovirology. 2009;6:1–12. 19493346 10.1186/1742-4690-6-54 PMC2700066

[38] Song H, Giorgi EE, Ganusov VV, Cai F, Athreya G, Yoon H, et al. Tracking HIV-1 recombination to resolve its contribution to HIV-1 evolution in natural infection. Nat Commun. 2018;9(1):1928. 29765018 10.1038/s41467-018-04217-5 PMC5954121

[39] Jiang J, Aiken C. Maturation-dependent human immunodeficiency virus type 1 particle fusion requires a carboxyl-terminal region of the gp41 cytoplasmic tail. J Virol. 2007;81(18):9999–10008. 17609279 10.1128/JVI.00592-07 PMC2045384

[40] Kalia V, Sarkar S, Gupta P, Montelaro RC. Rational site-directed mutations of the LLP-1 and LLP-2 lentivirus lytic peptide domains in the intracytoplasmic tail of human immunodeficiency virus type 1 gp41 indicate common functions in cell-cell fusion but distinct roles in virion envelope incorporation. J Virol. 2003;77(6):3634–46. 12610139 10.1128/JVI.77.6.3634-3646.2003 PMC149489

[41] Montero M, van Houten NE, Wang X, Scott JK. The membrane-proximal external region of the human immunodeficiency virus type 1 envelope: dominant site of antibody neutralization and target for vaccine design. Microbiol Mol Biol Rev. 2008;72(1):54–84. 18322034 10.1128/MMBR.00020-07 PMC2268283

[42] Wyma DJ, Jiang J, Shi J, Zhou J, Lineberger JE, Miller MD, et al. Coupling of human immunodeficiency virus type 1 fusion to virion maturation: a novel role of the gp41 cytoplasmic tail. J Virol. 2004;78(7):3429–35. 15016865 10.1128/JVI.78.7.3429-3435.2004 PMC371074

